# Zinc-finger proteins in health and disease

**DOI:** 10.1038/cddiscovery.2017.71

**Published:** 2017-11-13

**Authors:** Matteo Cassandri, Artem Smirnov, Flavia Novelli, Consuelo Pitolli, Massimiliano Agostini, Michal Malewicz, Gerry Melino, Giuseppe Raschellà

**Affiliations:** 1 Department of Experimental Medicine and Surgery, University of Rome ‘Tor Vergata’, Rome 00133, Italy; 2 Medical Research Council, Toxicology Unit, Leicester University, Leicester LE1 9HN, UK; 3 ENEA Research Center Casaccia, Laboratory of Biosafety and Risk Assessment, Via Anguillarese, Rome, Italy

## Abstract

Zinc-finger proteins (ZNFs) are one of the most abundant groups of proteins and have a wide range of molecular functions. Given the wide variety of zinc-finger domains, ZNFs are able to interact with DNA, RNA, PAR (poly-ADP-ribose) and other proteins. Thus, ZNFs are involved in the regulation of several cellular processes. In fact, ZNFs are implicated in transcriptional regulation, ubiquitin-mediated protein degradation, signal transduction, actin targeting, DNA repair, cell migration, and numerous other processes. The aim of this review is to provide a comprehensive summary of the current state of knowledge of this class of proteins. Firstly, we describe the actual classification of ZNFs, their structure and functions. Secondly, we focus on the biological role of ZNFs in the development of organisms under normal physiological and pathological conditions.

## Facts

- Zinc-finger proteins (ZNFs) are involved in several cellular processes acting through different molecular mechanisms.
- ZNFs have key role in development and differentiation of several tissues.
- ZNFs are involved in tumorigenesis, cancer progression and metastasis formation.
- Alterations in ZNFs are involved in the development of several of diseases such as neurodegeneration, skin disease and diabetes.

## Open questions

- ZNFs may act both as oncogene or tumor suppressor gene; can restoration or depletion of ZNFs expression be a new challenge in cancer drug design?
- Could ZNFs be used as a prognostic factor for cancer, neurodegeneration, or other diseases?

## ZNF structure, classification, and molecular functions

The first ZNF was identified in the late 1980s. The first ZNF was Transcription Factor IIIa (TFIIIa) from *Xenopus laevis.* This gave rise to the discovery of a new group of transcriptional activator proteins with a 30 amino acid repeating region. This new class of proteins was able to bind specific sequences of DNA.^1,2^ The zinc-finger structure (extensively reviewed in refs 3–7) is maintained by the zinc ion, which coordinates cysteine and histidine in different combinations. In classical C2H2 zinc-finger proteins, two cysteines in one chain and two histidines in other one are coordinated by a zinc ion. Crystallographic studies revealed that classical zinc-finger domains have two *β*-sheets and one α-helix.^8^

**Figure 1:**
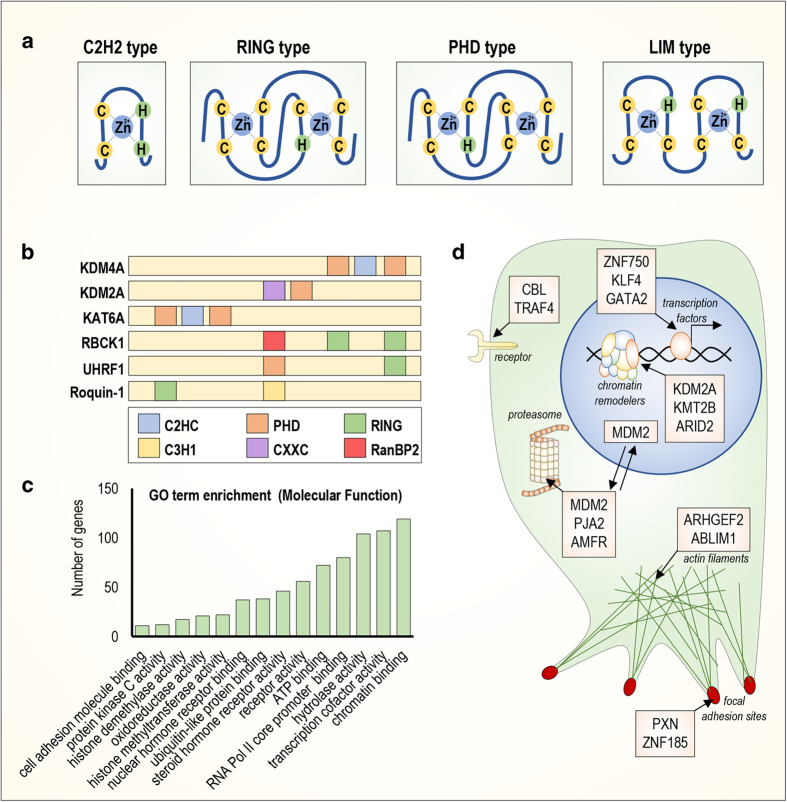
Structure, molecular functions, and subcellular localization of ZNFs. (**a**) A schematic representation of the structure of C2H2, RING, PHD, and LIM zinc-finger domains. (**b**) A schematic representation of the structure of some ZNFs with multiple zinc-finger domains. (**c**) Gene ontology analysis of 1723 annotated ZNFs according to molecular function, log_10_(*P*-val)<(−5). (**d**) Schematic representation of the subcellular localization of different ZNFs.

Non-classical types of zinc-finger differ in cysteine/histidine combinations, such as C2–H2, C2–CH, and C2–C2. Currently, 30 types of ZNFs are approved by The HUGO Gene Nomenclature Committee,^9^ and ZNF classification is based on the zinc-finger domain structure. A complete list of ZNF types with a description of the zinc-finger domain structure, the number of genes included, and the most studied members is summarized in Table 1. The most important and abundant types of zinc-finger domain proteins include C2H2,*** **really interesting new gene* (RING), *plant homeodomain* (PHD), and *Lin-ll, Isl-1, and Mec-3* (LIM domains). Their protein structures are presented in Figure 1a.

Among C2H2 ZNFs, there are a large number of transcription factors with the C-*x*-C-*x*-H-*x*-H motif, which mediates direct interaction with DNA. One of the C2H2 members, ZNF217, contains multiple C2H2 domains. These domains bind a specific DNA sequence (T/A)(G/A)CAGAA(T/G/C), repressing the expression of target genes.^10^

The group of RING domain proteins include numerous E3-ubiquitin ligases. The RING-motif structure is C-*x*-C-*x*-C-*x*-H-*x*-C-*x*-C-*x*-C-*x*-C. One of the most important E3-ubiquitin ligases, Mouse Double Minute 2 (MDM2), which is involved in cancer progression, has a RING domain. This domain mediates its interaction with itself and Mouse Double Minute 4 (MDM4). This domain is also important for E3-ubiquitin ligase activity.^11^

The PHD zinc-finger domains are involved in the regulation of epigenetic modifications via their chromatin-remodelling ability. The PHD motif has the following primary structure: C-*x*-C-*x*-C-*x*-C-*x*-H-*x*-C-*x*-C-*x*-C. One of the ZNFs with a PHD domain is Lysine Demethylase 2A (KDM2A), which mediates nucleosome recognition.^12^

The LIM-type of ZNF was identified in transcription factors *Lin-ll, Isl-1, and Mec-3*.^13^ Currently, this class of ZNFs contains proteins important for actin targeting, cytoskeleton interaction, and focal adhesion. One important member, Paxillin, has four LIM motifs. The LIM motif structure is C-*x*-C-*x*-H-*x*-C-*x*-C-*x*-C-*x*-C-*x*-(C,H,D).^14^ The zinc-finger domain of Paxillin mediates *β*-catenin interaction at focal adhesion sites^15^ and stress fibers.^16^

Interestingly, many ZNFs contain multiple and different types of zinc-finger domains (Figure 1b). For example, two lysine demethylases, Lysine Demethylase 4A (KDM4A), a novel target for antitumor therapy,^17^ and KDM2A, which is required for DNA damage response,^18^ exhibit different zinc-finger compositions. Nevertheless, an important acetyltransferase, Lysine Acetyltransferase 6A (KAT6A), which regulates cell cycle progression,^19^ has the same zinc-finger pattern as KDM4A but exhibits a different molecular function. Furthermore, RANBP2-Type and C3HC4-Type Zinc-Finger Containing 1 (RBCK1), Ubiquitin Like With PHD And Ring Finger Domains 1 (UHRF1), and Roquin-1 contain a RING-type zinc-finger domain that possesses E3-ubiquitin ligase activity. These proteins also possess additional different zinc-finger domains. For example, RBCK1 contains a RAN-binding protein 2 (RanBP2) domain and has an important role in the immune response.^20^ UHFR1 also contains a PHD domain that is important for its repressive activity on gene promoters.^21^ Finally, Roquin-1 possesses a C3H1 domain that targets RNA.^22^

Gene ontology analysis of 1723 annotated human ZNFs revealed that this class of proteins has numerous functions (Figure 1c). ZNFs localize in different cell compartments (Figure 1d). Indeed, chromatin-remodelling ZNFs (for example, KDM2A, Lysine Methyltransferase 2B (KMT2B) and AT-Rich Interaction Domain 2 (ARID2)) and transcription factors (for example, ZNF750, Kruppel Like Factor 4 (KLF4) and GATA Binding Protein 2 (GATA2)) are localized in the nucleus. Cbl Proto-Oncogene (CBL) and TNF Receptor Associated Factor 4 (TRAF4) are membrane proteins. MDM2, Praja Ring Finger Ubiquitin Ligase (PJA2), and Autocrine Motility Factor Receptor (AMFR) belong to the E3-ubiquitin ligase family are mainly localized in the cytoplasm. However, MDM2 has been also show to localize in the nucleus.^23,24^ Rho/Rac Guanine Nucleotide Exchange Factor 2 (ARHGEF2) and Actin-Binding LIM Protein 1 (ABLIM1) are associated with the cytoskeleton, and Paxillin with ZNF185 is localized to focal adhesion sites.^25^

The zinc-finger domain is one of the most frequently utilized DNA-binding motif found in eukaryotic transcriptional factors. The binding of a zinc-finger domain to its target site juxtaposes three base pairs on DNA to a few amino acids in the α-helix structure. The identity of the aminonoacids at the contact site defines the DNA sequence recognition specificity of zinc fingers. Thus, by changing these amino acids, a high degree of selectivity can be achieved toward a given three base-pair DNA sequence.^26^ Exploiting this recognition mechanism, protein modules containing multiple zinc-finger motifs, each one recognizing a specific three base-pair DNA sequence, have been engineered which bind to specific DNA sequences.^27^ Fusing this recognition module with a sequence-independent endonuclease was the first successful strategy to introduce breaks at specific sites of genomic DNA.^28^ Precise genome editing was more recently achieved with other techniques based on transcription activator-like effector nucleases (TALEN)^29^ and clustered regularly interspaced short palindromic repeats (CRISPR) Cas9^30^ whose description is beyond the scope of this review.

## Physiological role of ZNFs

### Skin

Through their ability to regulate gene expression, ZNF proteins participate in numerous physiological processes, including cell proliferation, differentiation, and apoptosis, thereby maintaining tissue homeostasis. For example, a recent study suggested that the zinc-finger proteins ZFP36 (RING-type, not transcription factor) an RNA binding protein, also known as Tristetrapolin^31^ and ZFP36L1 have a key role in the regulation of several aspects of keratinocyte biology, such as cell proliferation, differentiation and apoptosis. In fact, inhibition of the expression of these two proteins in cultured keratinocytes caused apoptosis and cell cycle arrest at the G2/M phase. In addition, *Zfp36* knockdown in these cells also results in increased expression of the differentiation marker Keratin 10, suggesting the possible involvement of this protein in keratinocyte differentiation.^32^

**Figure 2:**
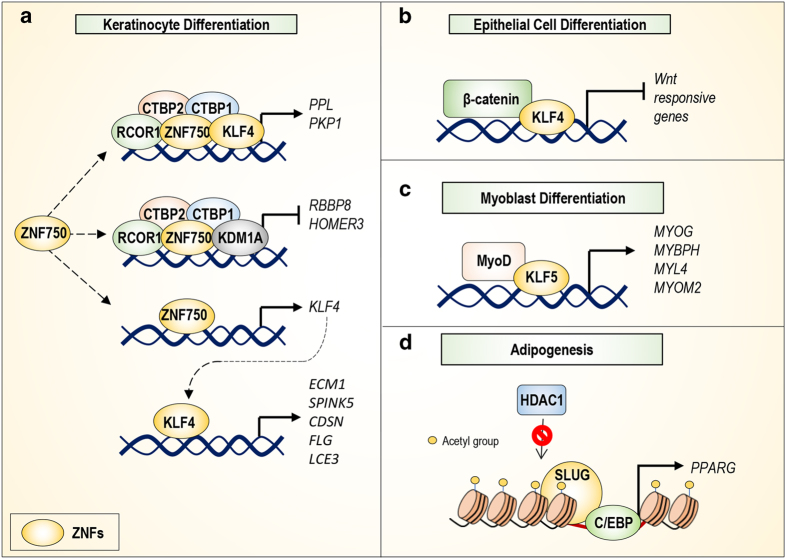
Molecular pathways regulated by ZNFs in physiological conditions. (**a**) ZNF750 regulates keratinocytes terminal differentiation by interacting with KLF4 and chromatin regulators. This interaction leads to the positive regulation of genes (*PPL, PKP1*) involved in differentiation. In addition, ZNF750 interacts with KDM1A and negatively regulates progenitor gene expression (*RBBP8, HOMER3*). ZNF750 directly regulates the expression of *KLF4*, which subsequently modulates the expression of the indicated genes. (**b**) KLF4 regulates epithelial cell differentiation by interacting with *β*-catenin and repressing the WNT signalling pathway. (**c**) KLF5 is involved in myoblast differentiation, acting as a co-factor for MyoD. This action leads to the upregulation of the indicated genes. (**d**) The presence of SLUG on the *PPARG* promoter reduces HDAC1 recruitment, leading to C/EBP-mediated activation of *PPARG* expression. This effect promotes adipogenesis.

Another zinc-finger protein that has a crucial role in keratinocyte differentiation, is the transcription factor, KLF4 (C2H2-type, transcription factor).^33^ Indeed, in the epidermis, KLF4 is mainly expressed in suprabasal layers, where it modulates the expression of genes involved in keratinocyte differentiation (*ECM1, SPINK5, CDSN, FLG*, and *LCE3*)^34^ (Figure 2a). In *Klf4*^*−/−*^ mice, the absence of this ZNF protein results in altered skin barrier formation, causing embryonic death soon after birth.^35^ Conversely, the ectopic expression of KLF4 in basal keratinocytes of transgenic mice epidermis accelerates the differentiation process, resulting in early epidermal barrier formation.^36^

The zing-finger protein ZNF750 (C2H2-type, transcription factor) also acts as an essential regulator of keratinocyte differentiation. During epidermal differentiation, *TP63* transcription regulates ZNF750 expression, which subsequently directly activates KLF4 expression.^37^ However, more recently, a layer of complexity has been added to the underlying mechanism by which ZNF750 regulates terminal differentiation of keratinocytes. ZNF750 represses the expression of progenitor genes (*RBBP8, HOMER3)* by interacting with the chromatin modifiers REST corepressor 1 (RCOR1), lysine demethylase 1A (KDM1A) and C-terminal binding protein 1/2 (CTBP1/2) and activates differentiation genes (*PPL, PKP1)* by interacting with RCOR1, KLF4, and CTBP1/2^38^ (Figure 2a). Interestingly, as described below, alteration of ZNF750 transcriptional regulation network during keratinocyte differentiation, caused by ZNF750 mutations, is involved in the development of diseases, such as psoriasis.^39,40^

### Intestine

ZNF proteins are also involved in intestinal epithelium biology. For example, in addition to its well-documented role in skin homeostasis, KLF4 also has a key role in the intestines. In this tissue, *KLF4* is expressed in the terminally differentiated epithelial cells (luminal surface) and goblet cells (crypts), where it promotes differentiation and inhibits proliferation.^41–44^ In particular, KLF4 represses intestinal epithelium proliferation by interacting with *β*-catenin and inhibiting *β*-catenin-mediated gene expression (Figure 2b). In addition, *Klf4*^*−/−*^ mice lack goblet cells, indicating that KLF4 has an essential role in goblet cell differentiation.^45^

Another Krüppel-like factor, KLF5 (C2H2-type, transcription factor), is crucial for regulation of proliferation in the intestinal epithelium, exerting an opposing function to KLF4.^42^ *KLF5*, which is expressed in basal epithelial cells of the crypts, is activated by the Wnt signalling pathway, promoting cell proliferation.^41^

The GATA family members GATA4 (GATA-type, transcription factor) and GATA6 (GATA-type, transcription factor) have important roles in differentiation and homeostasis of the small intestinal epithelium. GATA4 expression is detected in the proximal but not the distal small intestine, having an important role in the maintenance of jejunal-ileal specifications. Indeed, in the jejuna of inducible intestine-selective GATA4 knockout mice, the inactivation of *GATA4* results in downregulation of genes specifically expressed in the jejunum and increased expression of specific ileum genes.^46^ *GATA6* is expressed in the entire small intestine, where is required for intestinal proliferation, secretory cell differentiation and absorptive enterocyte gene expression.^47^

### Muscle

ZNFs have a regulatory function in muscle differentiation. For example, SET and MYND Domain Containing 1 (SMYD1), which is specifically expressed in striated muscle, acts as an essential regulator of myogenesis.^48^ *SMYD1* (MYND-type, not transcription factor) deletion impaired myoblast differentiation, decreasing myofibre formation and reducing muscle-specific gene expression. Moreover, inhibition of *KLF5* expression in cultured C2C12 myoblasts suppresses myotube formation, suggesting that this zinc-finger protein is required for myogenic differentiation. In particular, at a molecular level, KLF5 promotes myoblast differentiation into myotubes by recruiting MyoD to muscle-specific target genes (*MYOG, MYBPH, MYL4,* and *MYOM2)*^49^ (Figure 2c). Further examples of zinc-finger proteins involved in the regulation of skeletal myogenesis include CXXC Finger Protein 5 (*CXXC5*) and Early Growth Response 3 (*EGR3*). In fact, CXXC5 facilitates myocyte differentiation by positively regulating skeletal muscle differentiation genes,^50^ whereas EGR3 promotes myoblast proliferation by stimulating nuclear factor kappa B (NF-*к*B) signaling.^51^ By contrast, myogenic cellular differentiation is negatively regulated by murine zinc-finger Zfp637 (C2H2-type, not transcription factor). Although its transcriptional activity has not been fully investigated, Zfp637 overexpression inhibits differentiation and promotes proliferation of myoblasts, potentially regulating murine telomerase reverse transcriptase (mTERT) expression.^52^

### Adipose tissue

Recent studies revealed an increased number of ZNFs as key transcriptional regulators involved in adipogenesis.^53^ For example, ZNF638 (Matrin-type, not transcription factor) seems to positively regulate this process given that its expression increases during preadipocyte differentiation. Indeed, ectopic expression of *ZNF638* results in increased adipogenesis *in vitro.* On the other hand, inhibition of *ZNF638* expression decreases differentiation by inhibiting the expression of adipocyte-specific genes. Specifically, ZNF638 promotes adipogenesis by acting as a transcriptional co-factor of CCAAT/enhancer-binding protein (C/EBP) and results in the expression of peroxisome proliferator-activated receptor γ (*PPARG*), which regulates adipocyte differentiation.^54,55^

The transcription factor SLUG (C2H2-type, transcription factor) is also involved in adipocyte differentiation *in vitro and in vivo*.^56^ Indeed, *SLUG* knockout mice exhibit decreased white adipose tissue (WAT) mass compared with wild-type mice, whereas the WAT size is increased in Slug-overexpressing mice. Accordingly, *SLUG*-deficient mouse embryonic fibroblasts (MEFs) exhibit impaired adipogenesis compared with wild-type MEFs. SLUG potentially controls WAT development by affecting Histone deacetylase 1 (HDAC1) recruitment to the *PPARG* promoter, favouring a more accessible chromatin state for *PPARG* transcriptional activators (Figure 2d).

In contrast to ZNF638 and SLUG, the GATA transcription factors GATA2 (GATA-type, transcription factor) and GATA3 (GATA-type, transcription factor) act as negative regulators of adipocyte differentiation.^57^ Indeed, their expression is detected in preadipocytes and is decreased during differentiation. Consistently, the ectopic expression of GATA2 and GATA3 in preadipocytes inhibits their transition to adipocytes by binding to the *PPARG* promoter, inhibiting *PPARG* expression. In addition, GATA2 and GATA3 also interact with C/EBPα, and C/EBP*β*, suppressing their transcriptional activities. Both molecular mechanisms are required to negatively regulate adipogenesis.

### Cellular stemness regulation

In mice, Zfp281 (C2H2-type, transcription factor), the murine homolog of ZNF281, has an important role in regulating cellular stemness by binding the promoter of Nanog and inhibiting its transcription.^58^ Further work demonstrated that Nanog transcriptional repression requires the coordinated activity of NANOG itself and Zfp281, which recruits the NuRD repressor complex.^59^ Functionally, modulation of Nanog expression by Zfp281 is an efficient mechanism to fine-tune reprogramming activity in stem cells.

## Role of ZNFs in diseases

### Tumour suppressor and oncogenic functions of ZNFs

Recent findings have highlighted the importance of ZNFs in cancer onset and progression. The zinc-finger family includes both tumour suppressor genes and oncogenes.^60,61^ ZNFs are involved in all the principal pathways of cancer progression from carcinogenesis to metastasis formation. Furthermore, ZNFs are involved in cancer via their transcription factor function. In addition, emerging evidence indicates the importance of zinc-finger proteins as recruiters of chromatin modifiers or as structural proteins that regulate cancer cell migration and invasion.

#### ZNF281

**Figure 3:**
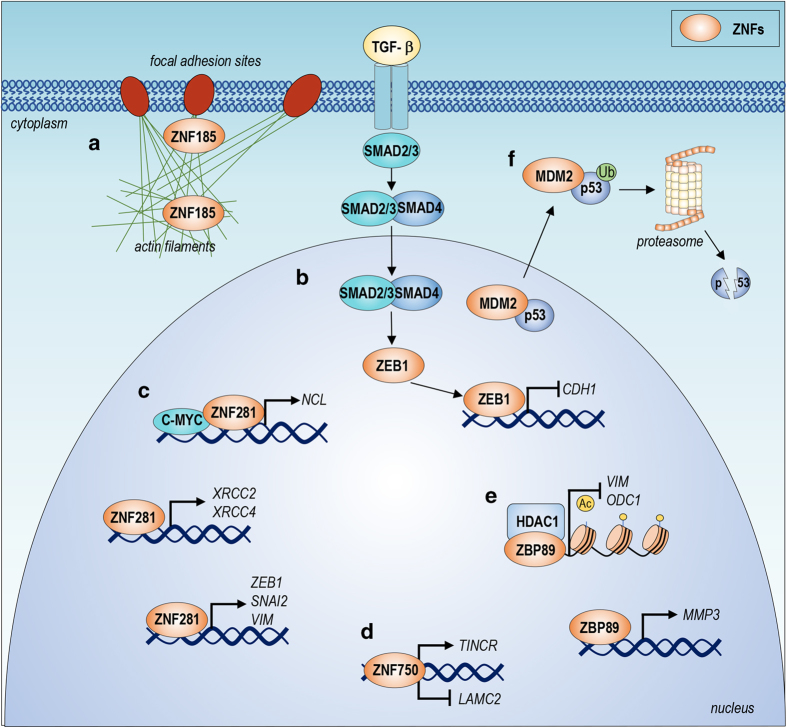
Molecular mechanisms underlying the role of ZNFs in cancer biology (**a**) ZNF185 interacts with actin filaments in focal adhesion sites to regulate migration and invasion. (**b**) TGF-*β* induces the expression of *ZEB1*, which represses *CDH1* expression, hence inducing EMT. (**c**) ZNF281 regulates expression of genes involved in the DDR and EMT. (**d**) ZNF750 acts as tumour suppressor gene by inducing the expression of the lncRNA *TINCR*, which inhibits cancer cell proliferation. In addition, ZNF750 represses *LAMC2* expression, inhibiting cancer cell migration. (**e**) ZBP89 represses *VIM* and *ODC1* expression by recruiting HDAC1 to the promoters of these genes. Moreover, ZBP89 induces *MMP3* expression. (**f**) MDM2 interacts with p53 to induce proteasomal degradation and impair p53 to exert its function.

In recent years, several experimental studies revealed a role of ZNF281 (C2H2-type) in tumorigenesis and tumour invasion. ZNF281 is involved in two crucial processes in cancer: the DNA damage response (DDR)^62–65^ and the epithelial–mesenchymal transition (EMT). *ZNF281* expression is increased upon DNA damage induced by drugs in several cancer types. In particular, the expression of several proteins involved in the DDR, including XRCC2, XRCC4, and Nucleolin, is regulated by ZNF281.^66^ Interestingly, two molecular mechanisms have been proposed: i) ZNF281 acts as transcription factor and directly regulates the transcription of XRCC2 and XRCC4; ii) ZNF281 also indirectly regulates Nucleolin expression, acting as a co-factor of c-Myc and producing an additive effect^46^ (Figure 3c).

**Figure 4:**
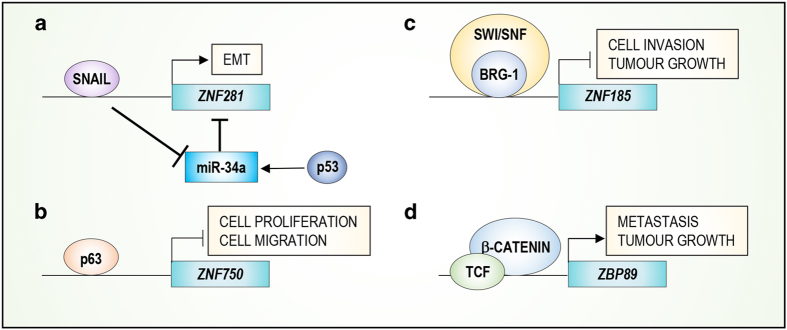
Transcriptional regulation of some ZNFs transcription and their roles in cancer. (**a**) SNAIL promotes the EMT by positively regulating the expression of *ZNF281* and negatively regulating the expression of the tumour suppressor miR-34a. (**b**) p63 induces *ZNF750* expression, which subsequently represses cell proliferation and migration. (**c**) *ZNF185* expression is regulated by Brg-1 and the SWI/SNF complex. Its activation represses cell invasion and tumour growth. (**d**) TCF/ *β*-catenin induces *ZBP89* expression, promoting tumour growth and metastasis.

Moreover, ZNF281 has also a role in metastasis in colorectal cancer (CRC) through regulation of the EMT^67^ (Figure 4a). During the EMT, *ZNF281* expression is induced by SNAIL and inhibited at the post-transcriptional level by miR-34a.^68–70^ The expression of miR-34a is subsequently promoted by p53, indicating that *ZNF281* in CRC is controlled by a feed-forward loop (Figure 4c). In addition, modulation of ZNF281 expression in CRC regulates the EMT through the activation of SNAIL expression. However, ZNF281 can also directly bind the promoters of EMT effector genes, such as *CDH-1*, *OCLN*, and *CLDN-7* (Figure 3c). Interestingly, ZNF281 expression was upregulated in patient tumour samples, confirming an important role of ZNF281 in CRC. These data strongly suggest that ZNF281 acts as an oncogene by regulating metastasis.

#### ZNF750

ZNF750 is another member of the family that is involved in cancer. Indeed, ZNF750 has been described as tumour suppressor gene in squamous cell carcinomas (SCCs) of the oesophagus, lung and cervix.^71,72^ ZNF750 is mutated in SCCs, and truncation and missense mutations represent the most common mutations. These mutations are located in the C2H2 zinc-finger domain, suggesting the importance of the zinc-finger domain in mediating the tumour suppressor activity of ZNF750. In addition, ZNF750 is expressed at much lower levels in SCC patients compared with normal tissue. Hence, ZNF750 overexpression *in vitro* inhibits cell proliferation and migration (Figure 4b). Interestingly, overexpression of the C2H2 ZNF750 mutant is not able to suppress tumour growth, demonstrating that the C2H2 zinc-finger domain is essential for the tumour suppressor activity of ZNF750. At the molecular level, ZNF750 regulates a set of genes involved in cell migration, proliferation and adhesion. Particularly, ZNF750 directly induces the expression of the long non-coding RNA TINCR, through which it regulates cancer cell proliferation and tumour growth and represses the expression of LAMC2, a component of Laminin-332. Collectively, these actions regulate cancer cell migration^73,74^ (Figure 3d). Accordingly, low expression of *ZNF750* has been observed in head and neck SCC and lung SCC patient datasets, and this expression pattern is associated with poor prognosis. Moreover, high levels of ZNF750 are associated with a good response to chemoradiotherapy, suggesting that ZNF750 could serve as a novel candidate biomarker for chemoradiotherapy sensitivity.^75^

#### ZNF185

ZNF185 (LIM-type, not transcription factor) is a zinc-finger protein that contains a LIM domain necessary for protein–protein interactions and an ATD (Actin Targeting Domain) domain with actin-binding activity.^76^ Proteins that contain LIM domains can be localized both in the nucleus and cytoplasm, exerting their molecular function through protein–protein interactions rather than DNA binding. The importance of ZNF185 in cancer progression is highlighted by its reduced expression in intermediate, high-grade, and metastatic prostate tumours compared with normal tissue. Interestingly, *ZNF185* expression is reduced in prostate cancer owing to DNA methylation. In fact, prostate cancer cell lines treated with a DNA Methyl Transferase 1 (DNMT1) inhibitor exhibit increased ZNF185 expression.^77^ Indeed, deregulation of ZNF185 expression seems to be a recurring event in different human cancers, including prostate cancer, primary lung tumours, colon cancer and HNSCC.^78,79^ These data suggest a putative tumour suppressor function for ZNF185 by regulating cell proliferation and differentiation.^80^ Moreover, in lung cancer, BRG1, a component of the human switch/sucrose non-fermenting complex (SWI/SNF), regulates *ZNF185* expression (Figure 4c). A possible mechanism by which ZNF185 exerts its function in cancer biology is through the interaction with actin filaments. Indeed, ZNF185 is associated with multiple actin-regulated structures, such as focal adhesion sites, and possesses growth inhibitory activity (Figure 3a). Localization of ZNF185 to the actin–cytoskeleton is mediated via its ATD domain, which is also required for its growth-suppressing activity. Furthermore, in prostate cancer, in addition to actin stress fibres, ZNF185 co-localizes with several cytoskeletal-related components, such as focal adhesion sites and filopodia/lamellipodia.^81^ These data suggest that ZNF185 may act as a novel tumour suppressor gene, having a key role in cancer onset and progression.

#### ZBP89

ZBP89 (C2H2-type, transcription factor), also known as ZNF148, is a well-characterized zinc-finger factor involved in cancer growth and apoptosis. Indeed, several tumours, such as breast cancer, melanoma and gastric cancer, exhibit increased ZBP89 expression compared with normal tissues, suggesting an oncogene function for *ZBP89*.^82–84^ However, *ZBP89* may act as tumour suppressor gene in colorectal cancer by repressing cell proliferation and inducing apoptosis.^85,86^ ZBP89 exerts its molecular function via two different mechanisms. First, it may act as autonomous transcription factor by regulating the expression of MMP3^87^ (Matrix Metallopeptidase 3) (Figure 3e) a protein involved in tumour development and metastasis.^88,89^ Second, ZBP89 inhibits ODC (Ornithine Decarboxylase)^90^ and Vimentin^91^ expression through recruitment of HDAC1 to the promoter of these genes^92,93^ (Figure 3e). ODC is involved in tumour development, and Vimentin has a role in cell migration and invasion. These findings suggest a role for ZBP89 in the inhibition of both neoplastic transformation and metastasis formation. Moreover, ZBP89 facilitates the recruitment of HDAC3 to the promoter of *CDKN2A* to restrain cellular senescence, facilitating lung cancer cell proliferation.^94^ Recently, it has been demonstrated that ZBP89 regulates the *β*-catenin pathway, supporting the hypothesis that ZBP89 is involved in cancer metastasis. Indeed, in colorectal cancer, the binding of ZBP89 to the promoter of *CTNNB* (*β*-catenin) results in increased gene expression. Interestingly, the inhibition of *β*-catenin expression resulted in a strong reduction in ZBP89 protein expression (Figure 4d). These data suggests that *β*-catenin accumulation initiates a cell proliferation program through the activation of its target genes, including Zbp89. Furthermore, the induction of ZBP89 contributes to sustaining *β*-catenin levels, further promoting cancer cell proliferation.^95^

#### MDM2

MDM2 (RANBP2-type; RING-motif, not transcription factor) is a zinc-finger protein that does not act as a transcription factor. Nevertheless, MDM2 has a very important role in tumour biology (extensively reviewed in Oliner *et al.*^96^). Its importance in cancer is attributed to its regulatory function on the tumour suppressor activity of p53. Indeed, MDM2 regulates p53 activity via three different mechanisms. First, given that MDM2 exhibits E3-ubiquitin ligase activity, it can ubiquitinate p53 to promote its proteasomal degradation. Second, MDM2 interacts with p53 to prevent the binding of p53 to its target genes, which mediate the tumour suppressor function of p53.^97–99^ Third, MDM2 binds to the N-terminus of p53, promoting the translocation of p53 into the cytoplasm and therefore blocking the activation of p53 target genes (Figure 3f).^100–102^ The importance of MDM2 in tumorigenesis is also provided by overexpression experiments. In fact, MDM2 overexpression induces spontaneous tumour formation.^103,104^ In addition, analysis of 28 tumour types performed on approximately 4000 patients revealed that the MDM2 gene is amplified in 7% of human cancers.^105^ Particularly, the percentage of MDM2 amplification is increased in liposarcomas (>80%), osteosarcomas (16%), soft tissue tumours (20%), and oesophageal carcinomas (13%).^106^ Moreover, point mutations affecting the zinc-finger of MDM2 have been described in human tumors.^107^ *In vitro* experiments show that these mutations disrupt the interaction of MDM2 with the ribosomal protein L5 and L11 and the ability to degrade p53.^108^ Given its importance, MDM2 is considered a putative target for therapies. An effort has been made to develop compounds that may prevent the interaction between MDM2 and p53, blocking the oncogenic activity of MDM2.^109^ As extensively reviewed by Wang *et al.*,^110^ three compounds (RG7112, RG7388 and SAR405838) exhibited relevant anti-tumoural activity in patients with p53 wild type in phase I clinical trials. Given that the anti-cancer activity of these compounds is attributed to the activation of wild type p53, and these compounds are expected be effective only in patients with wild type p53.^111^

#### ZEB1

ZEB1 (C2H2-type, transcription factor) is one of the most important zinc-finger proteins involved in tumour invasion and metastasis. Indeed, ZEB1 is one of the master regulators of the EMT^112^ (extensively reviewed in Zhang *et al.*^113^). *ZEB1* expression is regulated by several signalling pathways, such as Wnt, TGF-*β*, NF-*κ*B, and HIF signalling, and miRNA.^114^ The oncogenic role of ZEB1 is due to the repression of E-Cadherin expression, which is one of the most important cell–cell adhesion proteins. ZEB1 exerts its molecular function on E-Cadherin by interacting with several chromatin-remodelling factors, such as CtBP^115^ and the SWI/SNF complex.^116^ On the other hand, ZEB1 also directly activates the promoter of genes involved in the EMT. ZEB1 interacts with SMAD protein or with p300-P/CAF and activates TGF-*β* responsive genes to promote the EMT.^117,118^ Among ZEB1-activated genes, *CDH2* (N-cadherin), a mesenchymal cadherin, is important in cancer progression (Figure 3b) given that altered expression of ZEB1 is observed in several human cancers, including pancreatic cancer, lung cancer, liver cancer, osteosarcoma, breast cancer, and colon cancer.^109,119–122^ Furthermore, the overexpression of ZEB1 in several cancer lines induces the EMT and promotes cell invasion.^123,124^

### ZNF family members in neurodegenerative diseases

#### ZPR1

In recent years, ZNFs have been demonstrated to have an important role in the pathogenesis of neuronal diseases. Spinal muscular atrophy (SMA) is a rare neuromuscular disorder characterized by loss of α-motor neurons in the anterior horn of the spinal cord and progressive muscle wasting, often leading to early death.^125^ The cause of the disease is a mutation in the *Survival Motor Neurons 1* (*SMN1*) gene that results in reduced expression of the full-length SMN protein, which is necessary for survival of motor neurons.^126^ The first evidence of a possible involvement of ZPR1 (C4-type, not transcription factor) in SMA came from the experimental observation that the SMN protein interacts with ZPR1. The consequence of this interaction is a redistribution of the complex from the cytoplasm to the nucleus. Interestingly, this process is hampered in patients affected by SMA type I. In addition, this observation is also corroborated by evidence demonstrating that ZPR1 is expressed at low levels in patients with severe SMA.^127^ Furthermore, it has been reported that, mutation of *ZPR1* resulted in embryonic lethality in mice. Moreover, the reduction of *ZPR1* expression in mice, results in increased loss of spinal motor neuron, a similar phenotype observed in mice with reduced *Smn* gene, suggesting that the lower *ZPR1* expression observed in SMA patients, can contribute to the gravity of SMA.^128^

#### ZNF179

Zinc-Finger Protein 179 (ZNF179) (C4-type, not transcription factor) belongs to the RING finger class, and its expression is restricted in the brain, suggesting a possible role in the central nervous system.^129^ Indeed, inhibition of ZNF179 expression reduced neuronal differentiation in P19 cells and primary culture of cerebellar granule cells by inhibiting cell cycle progression through the regulation of p35 expression and the accumulation of p27 protein. More recently, it has been shown that ZNF179 has an anti-apoptotic role in astrocytes derived from the mouse APPtg model of Alzheimer’s disease. This effect is in part due to the inhibition of *IGFBP3* and *BIK* expression.^130,131^

#### ZNF746

**Figure 5:**
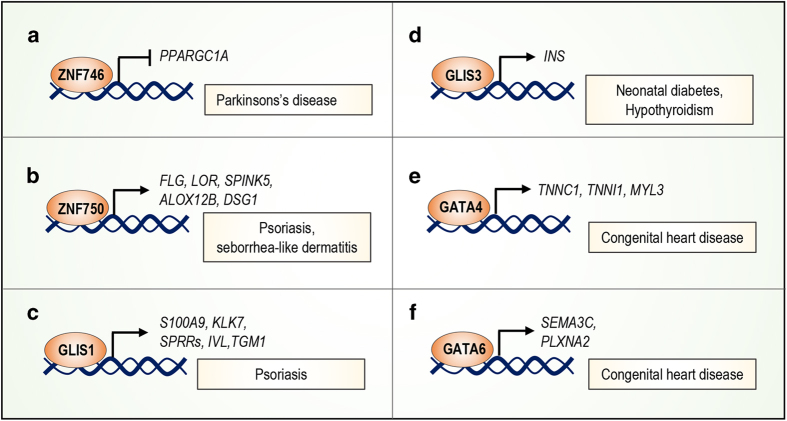
Regulation of ZNFs target genes in human diseases. (**a**) ZNF746 represses the expression of PGC-1α, resulting in the loss of dopaminergic neurons in the substantia nigra of Parkinson’s patients. (**b**) ZNF750 regulates the expression of epidermal differentiation markers, such as FLG, LOR, SPINK5, ALOX12B, and DSG, which are altered in human skin diseases. (**c**) Glis1 regulates transcription of several genes involved in the differentiation of epidermal keratinocytes, including cornifin, involucrin, and transglutaminase 1. The expression of these genes is altered in psoriasis. (**d**) Glis3 modulates expression of the insulin gene, contributing to the pathogenesis of neonatal diabetes and hypothyroidism. (**e**) Troponin C and I and myosin light chain-3 genes are induced during cardiac hypertrophy due to overexpression of the GATA4 transcription factor. (**f**) The expression of SEMA3C and its receptor PLXNA2 is downregulated by GATA6 mutations, resulting in the development of OFT defects associated with CHDs.

Recently, a novel role for ZNF746 (C2H2-type, not transcription factor), also known as Parkin Interacting Substrate (PARIS), has been identified in the pathogenesis of Parkinson’s disease (PD).^132^ Human ZNF746 is protein that contains C2HC and C2H2-type zinc-finger domains at the C-terminus. This protein is regulated by the proteasome system, in particular by ubiquitination mediated by Parkin, an E3-ubiquitin ligase. PD-associated mutations in the *PARK2* gene lead to the loss of its E3 ligase function, resulting in ZNF746 accumulation in human PD brain.^133^ ZNF746 overexpression results in the loss of dopaminergic neurons in the substantia nigra by repressing the expression of peroxisome proliferator-activated receptor gamma (PPARγ) coactivator-1α *(PPARGC1A)*^132^ (Figure 5a).

### ZNFs in other human diseases

#### ZNF750

Increasing evidence confirms the important roles of ZNFs in psoriasis. Psoriasis is a chronic inflammatory disorder of the skin, which varies in severity and clinical manifestation. ZNF750 is associated with a seborrhea-like dermatitis with psoriasiform elements.^40^ In particular, the 56_57dupCC mutation in *ZNF750* has been identified in psoriasis patients and results in a frameshift mutation. This mutation leads to the production of a truncated protein that does not contain the zinc-finger domain. Downregulation of *ZNF750* leads to reduced expression of genes involved in epidermal differentiation and skin barrier formation, such as *Filaggrin (FLG)*, loricrin (*LOR)*, serine protease inhibitor Kazal-type 5 (*SPINK5)*, Arachidonate 12-Lipoxygenase, 12R Type (*ALOX12B)* and desmoglein1 (*DSG1)* (Figure 5b). These genes are mutated in various human skin diseases. In fact, the clinical manifestations of skin diseases derived from *ZNF750* human mutations result from a combination of mutations in some of those downstream genes. ZNF750 and its downstream genes could be important targets for the treatment of skin diseases.^39,134^

#### GLIS1

Gli-similar protein 1 (GLIS1) (C2H2-type, transcription factor) is Krüppel-like zinc-finger protein involved in the pathogenesis of psoriasis. Indeed, *GLIS1* is significantly overexpressed in psoriatic epidermis.^135^ *GLIS1* mRNA is present only in the suprabasal layers of psoriatic skin, whereas normal human epidermis does not express GLIS1. These data suggest that GLIS1 that could be involved in the regulation of abnormal differentiation observed in psoriatic epidermis. Consistently, microarray analysis reveals that ectopic expression of GLIS1 transcriptionally regulates the expression of several genes involved in the differentiation of epidermal keratinocytes, including *S100A9*, *KLK7*, small proline-rich proteins (*SPRRs*), involucrin (*IVL)*, and transglutaminase 1 (*TGM1*) (Figure 5c). GLIS1 contains both a repressor domain at its amino terminus and an activation domain at its carboxy terminus, resulting in both transcriptional repressor and transactivator functions. GLIS1 regulates transcription of target genes through binding to oligonucleotides containing the Gli-binding site consensus sequence, GACCACCCAC, as demonstrated by electrophoretic mobility shift assays. In addition, GLIS1 is expressed in different temporal and spatial patterns during the embryonic development, thus regulating gene expression at different stages of the developmental process as demonstrated by whole mount *in situ* hybridization studies performed on mouse embryos.^136^

#### GLIS3

Several reports indicate that the ZNF family might have a role in the development of diabetes. For example, GLIS3 (C2H2-type, transcription factor), a member of Kruppel-like Zinc-Finger proteins, is highly expressed in human pancreatic *β*-cells, and mutations in the *GLIS3* gene have been identified in neonatal diabetes and congenital hypothyroidism (NDH).^137^ A human *GLIS3* mutation that results in a truncated protein at its C-terminal domain has been identified, but the specific mechanism by which this mutation leads to the development of NDH has not been investigated to date. GLIS3 modulates the expression of the insulin through both direct and indirect mechanisms: binding to the *INS* promoter (Figure 5d) or modulating the activity of other *β*-cell-enriched transcription factors, such as MafA, Nkx6-1, and Pax6. Recently, a GLIS3-deficient (*Glis3*^−/^^−^) mouse model has been generated, exhibiting high blood sugar levels, pancreatic defects and premature death. These phenotypes resemble human neonatal diabetes caused by *GLIS3* mutations.^138^ This murine model could be very useful for studying novel therapeutic applications in human diabetes. Novel clinical manifestations for patients with neonatal diabetes caused by *GLIS3* mutations have been identified, such as osteopenia associated with skeletal deformity and fractures, bilateral sensorineural deafness and exocrine pancreatic dysfunction. These clinical features were not previously described, demonstrating great variability in *GLIS3* mutated phenotype given that different genetic mutations result in tissue-specific expression of *GLIS3* mRNA.^139^

#### GATA4

ZNFs are involved also in the pathogenesis of congenital heart diseases (CHDs). CHDs are the most common developmental anomaly affecting new-borns. For example, GATA4 is essential for proper cardiac morphogenesis. Indeed, *GATA4* mutations are implicated in human congenital heart disease. A heterozygous G296S missense mutation of *GATA4* has been identified^140^ that causes reduced transcriptional activity and DNA-binding affinity of GATA4. Furthermore, the *GATA4* mutation prevents the physical interaction between GATA4 and TBX5, a T-box protein responsible for a subset of syndromic cardiac septal defects.^141,142^ Overexpression of GATA4 is associated with cardiac hypertrophy, where directly it regulates the expression of several cardiac specific proteins, such as troponin C and I and myosin light chain-3 (Figure 5e). Interestingly, these genes are induced during cardiac hypertrophy.^143^ In addition, expression of several other proteins, including Na+/Ca^2^+-exchanger, acetylcholine receptor-M2, cardiac-restricted ankyrin repeat protein (CARP), and adenosine receptor-A1 and carnitine palmitoyltransferase-1*β*, is regulated by GATA4.^144,145^ These findings suggest that GATA transcription factors could be an attractive therapeutic target for the treatment of cardiovascular diseases.

#### GATA6

Another GATA zinc-finger transcription factor expressed in the developing heart is GATA6. Mutations in this gene have been identified in patients with CHDs.^146^ Recent studies demonstrated that downregulation of *GATA6* in neural crest-derived smooth muscle causes defects of the cardiac outflow tract (OFT) and in aortic arch arteries.^147,148^ GATA6 regulates neurovascular guiding molecule semaphorin 3C (*SEMA3C*) and its receptor plexin A2 (*PLXNA2*) expression (Figure 5f), which is important for a normal OFT. *GATA6* mutations result in downregulation of these genes, disrupting semaphorin–plexin signalling and contributing to OFT defects, which accounts for 30% of CHDs.

Similar to GATA4, GATA6, and Tbx5 are co-expressed in the embryonic heart, and their interaction is necessary to activate the atrial natriuretic factor promoter during cardiac morphogenesis. The interaction between the GATA family of transcription factors and Tbx5 is necessary for proper cardiac function. Indeed, mutations in *GATA4*, *GATA6*, and *TBX5* genes disrupt these interactions, contributing to the pathogenesis of CHDs.^149^

These data contribute to the identification of *GATA* mutations as a major genetic cause of CHDs.

## Conclusions

It is now well accepted that ZNFs have a crucial role both in tissue homeostasis and disease. Interestingly, although this class of proteins was initially classified as transcription factors, several studies have highlighted novel functions of ZNFs. In fact, it has been shown that ZNFs could also act as recruiters of chromatin modifiers, as co-factors, or as structural proteins involved in cell migration and invasion.

In particular, the role of ZNFs in cancer development, progression and metastasis is becoming an interesting research issue. In fact, ZNF expression is upregulated or downregulated in cancer patients, demonstrating that ZNFs may act both as tumour suppressors or oncogenes. Furthermore, the functions of several ZNFs seem to be selective for specific tumours. Thus, the design of drugs that target specific ZNFs to avoid or restore abnormal expression of these proteins could be one of the most important challenges in the near future. Moreover, given the high specificity in terms of function and expression of some ZNFs for some tumours, it could be useful to exploit this class of proteins as prognostic factors.

## Additional information

**Publisher’s note:** Springer Nature remains neutral with regard to jurisdictional claims in published maps and institutional affiliations.

## Figures and Tables

**Table 1:** Types of zinc-finger proteins

*Type name*	*Zinc-finger structure*	*Number of genes*	*Number of TF*	*Important members*
Zinc fingers C2H2-type (ZNF)	C-x-C-x-H-x-H	720	372	*KLF4, KLF5, EGR3, ZFP637, SLUG, ZNF750, ZNF281, ZBP89, GLIS1, GLIS3*
Ring finger proteins (RNF)	C-x-C-x-C-x-H-xxx-C-x-C-x-C-x-C	275	12	*MDM2, BRCA1, ZNF179*
PHD finger proteins (PHF)	C-x-C-x-C-x-C-xxx-H-x-C-x-C-x-C	90	0	*KDM2A, PHF1, ING1*
LIM domain containing	C-x-C-x-H-x-C-x-C-x-C-x-C-x-(C,H,D)	53	1	*ZNF185, LIMK1, PXN*
Nuclear hormone receptors (NR)	C-x-C-x-C-x-C-xxx-C-x-C-x-C-x-C	50	47	*VDR, ESR1, NR4A1*
Zinc fingers CCCH-type (ZC3H)	C-x-C-x-C-x-H	35	2	*RC3H1, HELZ, MBNL1, ZFP36, ZFP36L1*
Zinc fingers FYVE-type (ZFYVE)	C-x-C-x-C-x-C-xxx-C-x-C-x-C-x-C	31	0	*EEA1, HGS, PIKFYVE*
Zinc fingers CCHC-type (ZCCHC)	C-x-C-x-H-x-C	25	2	*CNBP, SF1, LIN28A*
Zinc fingers DHHC-type (ZDHHC)	C-x-C-x-H-x-C-xxx-C-x-C-x-H-x-C	24	0	*ZDHHC2, ZDHHC8, ZDHHC9*
Zinc fingers MYND-type (ZMYND)	C-x-C-x-C-x-C-xxx-C-x-C-x-H-x-C	21	4	*PDCD2, RUNX1T1, SMYD2,SMYD1*
Zinc fingers RANBP2-type (ZRANB)	C-x-C-x-C-x-C	21	3	*YAF2, SHARPIN, EWSR1*
Zinc fingers ZZ-type (ZZZ)	C-x-C-x-C-x-C	18	3	*HERC2, NBR1, CREBBP*
Zinc fingers C2HC-type (ZC2HC)	C-x-C-x-H-x-C	16	2	*IKBKG, L3MBTL1, ZNF746*
GATA zinc-finger domain containing (GATAD)	C-x-C-x-C-x-C	15	15	*GATA4, GATA6, MTA1*
ZF class homeoboxes and pseudogenes	C-x-C-x-H-x-H	15	10	*ADNP, ZEB1, ZHX1*
THAP domain containing (THAP)	C-x-C-x-C-x-H	12	3	*THAP1, THAP4, THAP11*
Zinc fingers CXXC-type (CXXC)	C-x-C-x-C-x-C-xxx-C-x-C-x-C-x-C	12	2	*CXXC1, CXXC5, MBD1,DNMT1*
Zinc fingers SWIM-type (ZSWIM)	C-x-C-x-C-x-H	9	0	*MAP3K1, ZSWIM5, ZSWIM6*
Zinc fingers AN1-type (ZFAND)	C-x-C-x-C-x-C-xxx-C-x-H-x-H-x-C	8	0	*ZFAND3, ZFAND6, IGHMBP2*
Zinc fingers 3CxxC-type (Z3CXXC)	C-x-C-x-H-x-C	8	0	*ZAR1, RTP1,RTP4*
Zinc fingers CW-type (ZCW)	C-x-C-x-C-x-C	7	0	*MORC1, ZCWPW1,KDM1B*
Zinc fingers GRF-type (ZGRF)	C-x-C-x-C-x-C	7	0	*TTF2, NEIL3, TOP3A*
Zinc fingers MIZ-type (ZMIZ)	C-x-C-x-H-x-C	7	1	*PIAS1, PIAS3, PIAS4*
Zinc fingers BED-type (ZBED)	C-x-C-x-H-x-H	6	2	*ZBED1, ZBED4, ZBED6*
Zinc fingers HIT-type (ZNHIT)	C-x-C-x-C-x-C-xxx-C-x-C-x-H-x-C	6	0	*ZNHIT3, DDX59, INO80B*
Zinc fingers MYM-type (ZMYM)	C-x-C-x-C-x-C	6	6	*ZMYM2, ZMYM3, ZMYM4*
Zinc fingers matrin-type (ZMAT)	C-x-C-x-H-x-H	5	0	*ZNF638, ZMAT1, ZMAT3, ZMAT5*
Zinc fingers C2H2C-type	C-x-C-x-H-x-H	3	3	*MYT1, MYT1L, ST18*
Zinc fingers DBF-type (ZDBF)	C-x-C-x-H-x-H	3	0	*DBF4, DBF4B, ZDBF2*
Zinc fingers PARP-type	C-x-C-x-H-x-C	2	1	*LIG3, PARP1*
